# Microbial and periodontal shifts induced by orthodontic appliances: a comparative clinical study

**DOI:** 10.2340/aos.v85.45321

**Published:** 2026-01-20

**Authors:** Aybüke Asena Atasever İşler, Abdulvahit Erdem

**Affiliations:** a Assistant Professor, Department of Orthodontics, Faculty of Dentistry, Bolu Abant Izzet Baysal University, Bolu, Turkey; b Professor, Department of Orthodontics, Faculty of Dentistry, Kafkas University, Kars, Turkey

**Keywords:** Appliances, oral microbiota, periodontics, VITEK 2

## Abstract

**Objective:**

This study aimed to evaluate the periodontal and microbiological alterations in oral flora associated with using removable appliances and fixed orthodontic treatments.

**Methods:**

The study materials consist of subgingival-supragingival plaque samples and periodontal measurements from 48 patients. The groups include fixed appliance users, removable appliance users, and a nontreatment control group. Periodontal measurements, including gingival index, plaque index, bleeding on probing, mobility, furcation, probing depth, and attachment level, were recorded at baseline (before treatment) and 6 months after treatment initiation. Plaque samples were collected at T0 and T1. Dental plaque samples were cultured on selective media for qualitative and quantitative microbial analysis, followed by qualitative evaluation using the VITEK 2 (Biomerieux) system. Data were analyzed using one-way one-way analysis of variance one-way analysis of variance (ANOVA), paired sample-T test, and Duncan’s multiple range test to identify and compare statistically significant differences within and between groups, while intraobserver reliability was assessed using the Kappa statistic.

**Results:**

Mobility or furcation involvement was not detected in any of the groups at either time point. At the T1 stage, a slight improvement in attachment level was observed in both the fixed and removable appliance groups compared with baseline measurements. However, during the same period, a significant increase in mean probing depth was detected only in the fixed appliance group (*p* = 0.003). Plaque index levels increased in both the removable appliance group (*p* = 0.019) and the fixed appliance group (*p* = 0.023). Furthermore, the bleeding on probing index also showed an increase in both groups, with *p* = 0.020 in the removable appliance group and *p* = 0.012 in the fixed appliance group. At the T1 stage, an increase in yeast counts was observed in the removable appliance group (*p* = 0.008), whereas decreases were detected in *Lactobacillus* (*p* = 0.004) and mutans streptococci (*p* = 0.026) levels. In contrast, the fixed appliance group demonstrated significant increases in *Lactococcus* (*p* = 0.042) and mutans streptococci (*p* = 0.037) counts. The identified microorganisms included a diverse range of bacterial species, such as *Actinomyces spp., Fusobacterium spp., Lactobacillus spp., Streptococcus spp., Veillonella spp.,* and other clinically significant genera.

**Conclusions:**

It may be inferred that fixed orthodontic treatments create a biological environment that is more susceptible to adverse periodontal alterations and increased colonization by specific microbial species. Conversely, the potential suppressive effects of removable appliances on certain microbial groups emphasize the need to consider patients’ oral hygiene compliance, periodontal risk profile, and microbial sensitivity during treatment planning.

## Introduction

The primary objectives of orthodontic treatment are to establish optimal occlusion, enhance facial esthetics, and ensure long-term stability while preserving the health of periodontal structures. Conventional fixed orthodontic appliances continue to serve as a widely preferred treatment modality in contemporary orthodontic practice, with their clinical effectiveness supported by scientific evidence [1]. Both fixed and removable orthodontic appliances, including bands, brackets, and clasps, can promote plaque accumulation in the areas surrounding the associated teeth [2]. Using bands and brackets in orthodontic treatments facilitates the retention of food particles, leading to decreased pH, increased colonization of *Streptococcus mutans* and *Lactobacillus acidophilus*, biofilm formation, and plaque accumulation. These conditions contribute to the development of white spot lesions, which include enamel decalcification and cavitation, potentially resulting in irreversible damage. *S. mutans* is the primary microorganism implicated in the progression of dental caries [3, 4].

A study [5] has shown that orthodontic attachments substantially elevate the prevalence of cariogenic bacteria, especially *Streptococcus mutans*, with bacterial counts increasing up to fourfold compared to the beginning of treatment. Moreover, one study [6] reported the presence of nonoral opportunistic pathogens, including *Staphylococcus* and *Candida* species, on orthodontic retainers and the oral mucosa. Furthermore, fixed orthodontic appliances have been reported to promote the growth of periodontopathogenic bacterial species, including *Porphyromonas gingivalis*, *Prevotella intermedia*, *Tannerella forsythia, Aggregatibacter actinomycetemcomitans*, *Fusobacterium nucleatum*, and *Treponema denticola* [7]. Taken together, these findings suggest that fixed orthodontic therapy may shift the oral microbiome toward a dysbiotic profile, thereby increasing the risk of plaque-induced soft-tissue complications. Gingival overgrowth associated with orthodontic therapy has been linked to inadequate oral hygiene, primarily resulting from bacterial plaque accumulation [8], tartar buildup [9], and the deposition of chromium and nickel released from orthodontic alloys [10] all of which may contribute to an inflammatory periodontal response. From a clinical perspective, early identification of adverse periodontal or microbial changes may allow timely preventive strategies, improving long-term prognosis and reducing the likelihood of irreversible sequelae such as gingival recession, bone loss, or treatment relapse.

Although the relationship between orthodontic appliances and periodontal health has been widely explored in previous clinical studies [1, 7, 8, 11–13], existing research has predominantly focused either on fixed appliances or removable appliances separately, often without employing a simultaneous comparative design or integrating both periodontal and microbiological outcomes at multiple time points. In contrast, the present study incorporates both fixed and removable orthodontic appliances within the same methodological framework, enabling direct comparison under identical clinical conditions, while also performing parallel periodontal and microbiological evaluations. Beyond this, the use of the VITEK 2 system provides a higher-resolution identification of microbial profiles, including less commonly detected taxa, thereby enhancing the microbiological diagnostic depth rather than serving merely as a novel tool. Therefore, this study aims to address an unmet gap in the literature by combining dual appliance comparison, longitudinal periodontal assessment, and advanced microbial identification to offer a more comprehensive understanding of how orthodontic treatment modalities influence oral microbial ecology and periodontal health.

The hypothesis of this study is that the use of fixed and removable orthodontic appliances leads to significant differences in periodontal parameters and oral microbial profiles, with fixed appliances resulting in a more unfavorable microbial and periodontal outcome.

## Materials and methods

### Study design

This comparative clinical study protocol was prepared by the Declaration of Helsinki. Ethical approval was obtained from Atatürk University Faculty of Dentistry Ethics Committee (Decision number: 57). Written informed consent forms were also obtained from the parents of the individuals participating in the study. We recruited 48 voluntary participants (30 females, 18 males), with an average age of 15.5 years, seeking orthodontic treatment at the Department of Orthodontics, Faculty of Dentistry, Atatürk University. The study sample comprised patients with mild dental crowding (2–3 mm), and all participants were selected based on comparable baseline periodontal characteristics to maintain methodological integrity and reduce potential bias. Based on their planned treatment modalities, the participants were assigned to either fixed appliance, removable appliance, or untreated control groups. Subgingival and supragingival dental plaque samples were collected from these participants at two distinct time points. Considering evidence indicating that [14] bracket material and design can influence enamel demineralization severity, Gemini Metal brackets (3M Unitek Monrovia, CA, USA) were selected for the current study due to their more favorable demineralization profile compared with ceramic and self-ligating alternatives. To ensure methodological standardization, all patients were treated with 0.018-inch Roth prescription brackets, and stainless steel wire ligatures were used for archwire engagement. Removable appliances, including expansion, distalization, and protrusion devices, were sanitized, fitted, and adjusted for proper occlusion, with detailed instructions provided to the patients. Moreover, to minimize operator-related variability, all appliances were delivered by a single clinician (A.A.A.İ).

The groups are defined as follows:

- Fixed appliance users (*n* = 16)
- Removable appliance users (*n* = 16)
- Nontreatment control group (*n* = 16)

Inclusion criteria:

- Age between 12 and 19 years,
- Normal root development,
- No previous history of orthodontic treatment.

Exclusion criteria:

- Presence of any missing teeth,
- Periodontal bone loss,
- Habit of nail-biting,
- Any systemic or syndromic diseases (e.g. hepatic, renal, hematological, cardiovascular),
- Congenital, genetic, or acquired craniofacial deformities due to trauma, such as cleft palate or lip,
- Smoking,
- Untreated tooth decay.

### Measurement of periodontal parameters

Periodontal evaluation was conducted using an online periodontal chart (https://www.periodontalchart-online.com) and categorized based on periodontal health, gingivitis, and periodontitis (stage and grade). All periodontal index measurements were performed by the corresponding author, who had received prior training from a periodontologist in the use and calibration of the indices applied in this study.

### Mobility assessment

Mobility was assessed manually through horizontal and vertical measurements. Horizontal mobility was evaluated using two hand instruments placed on the buccal and lingual surfaces, while vertical mobility was assessed by applying force perpendicular to the occlusal surface. The degree of mobility, if present, was measured using a Periotest device (Medizintechnik Gulden, Modautal, Germany) [15].

### Furcation assessment

Furcation involvement was assessed through clinical and radiographic examinations. Clinically, a Nabers probe was directed apically toward the furcation entrance and horizontal displacement into a space indicated furcation involvement [16].

### Clinical attachment level assessment

The clinical attachment level measures the distance from the base of the pocket to a fixed point on the crown, typically the cementoenamel junction. The gingival margin is first identified, representing the distance from the gingival boundary to a reference point, such as the cementoenamel junction, unless restoration margins apical to this junction are used. Probing depth refers to the distance from the gingival margin to the base of the gingival sulcus or periodontal pocket [17].

### Probing depth assessment

A periodontal probe (UNC 15, Hu-Friedy, USA) was used to measure probing depth, recording millimetric values for each tooth. The probe was aligned with the tooth’s long axis, and probing ceased upon encountering optimal resistance. The distance from the base of the pocket/sulcus to the gingival margin was measured at the mesiobuccal, distobuccal, midbuccal, and midpalatal surfaces. The mean probing depth was calculated by averaging the measurements from all recorded surfaces [18].

### Plaque index assessment

The plaque index was evaluated using the Silness and Löe method [19]. Each tooth surface was scored individually, and the total score was averaged by dividing it by four. Teeth were isolated with cotton rolls, and plaque presence and severity were assessed using an examination probe. The plaque index was calculated by dividing the total score by the number of teeth.

### Bleeding on the probing index assessment

The bleeding on the probing index was assessed by gently inserting a periodontal probe into the pocket and evaluating bleeding after approximately 20 s. Bleeding was recorded as (+) if present and (–) absent. The percentage of bleeding sites was calculated by dividing the number of bleeding sites by the total number of sites examined [20].

### Sample collection

Subgingival and supragingival dental plaque samples were collected from 48 volunteer participants in the study at two different time points:

**T0:** Before treatment.

**T1:** After 6 months of treatment.

**Figure 1:**
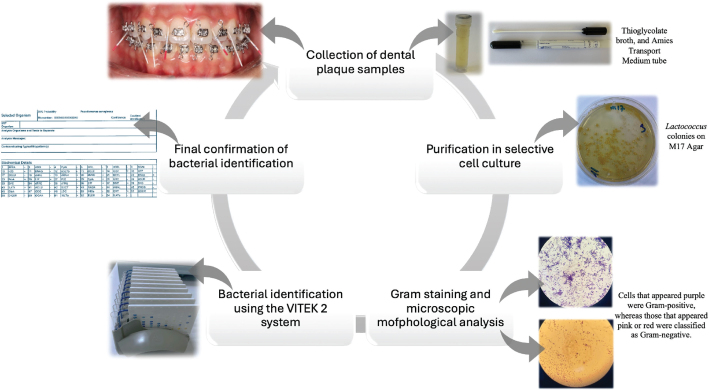
Isolation and identification of microorganisms.

Before subgingival sampling, all participants underwent professional supragingival plaque removal to prevent contamination. Subgingival plaque samples were collected from shallow sulci (2–3 mm probing depth) using sterile size #4 paper points, which were inserted for 20 s without causing gingival damage. Samples collected from the vestibular surfaces of 24 teeth using a periodontal probe were transferred to Amies transport medium tubes, whereas samples obtained with paper points were placed into tubes containing 2 ml of thioglycolate broth. All plaque samples were transported to the microbiology laboratory within 30 min under cold chain conditions to preserve microbial viability. Prior to culturing, tenfold serial dilutions (10^–^¹ to 10^–^³) were performed to ensure proper colony separation and minimize overgrowth, facilitating accurate isolation and identification of pure colonies. Additionally, supragingival dental plaque samples were collected from the vestibular surfaces of 24 teeth using a scaler and transferred in 2 ml of tryptic soy broth for microbial analysis. Cells that appeared purple were Gram positive, whereas those that appeared pink or red were classified as Gram negative. The morphological characteristics of the microorganisms, including their size, shape, and arrangement, were examined on Gram-stained slides [21] (Figure 1).

### Microbiological analysis

**Figure 2:**
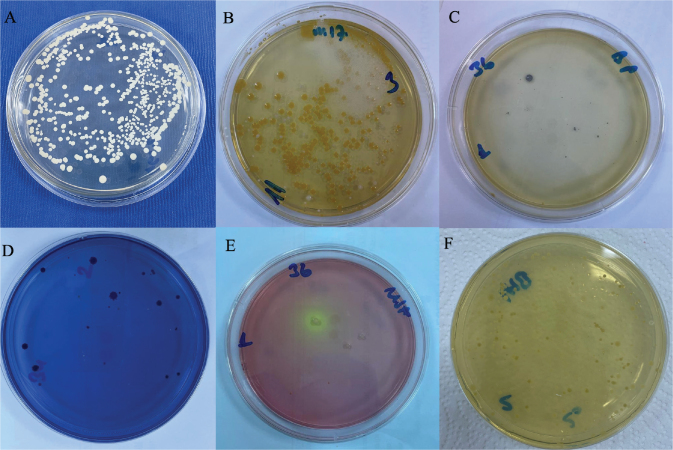
Yeast colonies on Sabouraud Dextrose Agar (A), *Lactococcus* colonies on M17 Agar (B), *S. aureus* colonies on Baird Parker Agar (C), *S. mutans* colonies on Mitis Salivarius-Bacitracin Agar (D), *Staphylococcus*-*Micrococcus* colonies on Mannitol Salt Agar (E), and total anaerobic bacterial colonies on Brain Heart Infusion Agar (F).

All microbiological procedures were also carried out by the corresponding author with support from a microbiology expert. The number of microorganisms was determined through quantitative analysis, which involved counting colony-forming units (CFU) per milliliter of solution. The researcher applied a spread plate technique to distribute 100 µL of each dilution across selective and nonselective agar media after performing serial dilutions from 10^–^¹ to 10^–^³ (Figure 2). The digital colony counter enabled researchers to count colonies between 30 and 300 per plate after the plates received their required aerobic or anaerobic incubation conditions. The CFU/ml values were determined through the following calculation: CFU/ml = (number of colonies × dilution factor) / plated volume (ml). The research team documented their microbial count results as average CFU/ml values for each tested sample.

### Identification of bacteria using the VITEK 2 (bioMérieux, Hazelwood, MO, USA) system

Anaerobic conditions were created using AnaeroGen™ (Oxoid Ltd., Basingstoke, UK) sachets in anaerobic jars to achieve a gas mixture of 10% CO₂, 10% H₂, and 80% N₂. The absence of oxygen was verified with anaerobic indicators. Furthermore, bacterial species grown on anaerobic media were not automatically assumed to be obligate anaerobes. After colony isolation, identification with VITEK 2 (bioMérieux, Hazelwood, MO, USA) allowed differentiation between obligate and facultative anaerobes, ensuring accurate classification.

The VITEK 2 system is an automated microbial identification and antibiotic susceptibility testing platform widely used in clinical microbiology laboratories [22], offering a practical and rapid solution for routine microbial identification. It works by analyzing the biochemical and metabolic characteristics of organisms using miniaturized tests with fluorescence-based detection, quickly determining the organism’s identity to the species level [23]. In practice, VITEK 2 can deliver identification results within a few hours, which is a major advantage compared to 16S rRNA gene sequencing that may take several days despite its high accuracy. While MALDI-TOF MS can identify cultured bacteria within minutes, it does not simultaneously perform susceptibility testing and requires an expensive mass spectrometer instrument [24]. By contrast, VITEK 2 offers an integrated, fully automated workflow that provides both identification and antibiotic susceptibility results in a short time, making it highly effective for routine clinical use.

The VITEK 2 system uses separate cards to identify and test the susceptibility of Gram-positive and Gram-negative microorganisms. Each gram-positive (GP) and gram-negative (GN) card contains 64 wells, enabling simultaneous identification and antibiotic susceptibility testing of up to 60 isolates. The system performs 43 biochemical tests, assessing carbon source utilization, enzymatic activity, and resistance. After card filling, the system automatically transfers the cards to the reader incubator, takes optical readings, and discards the cards post-analysis. Results are typically available within 8 h [25].

Bacterial identification was performed for aerobic, microaerophilic, and anaerobic bacteria using specific culturing and testing methods. Aerobic bacteria were isolated by inoculating samples into tryptic soy broth, plating onto selective agar media (Plate Count Agar, Blood Agar, MacConkey Agar, and Mannitol Salt Agar), and incubating at 37°C for 24–48 h. Microaerophilic bacteria were cultured on Brain Heart Infusion Agar supplemented with vitamin K and haemin under microaerophilic conditions (5–10% CO₂) for 2–7 days, while anaerobic bacteria were grown on Reinforced Clostridial Medium, 5% Sheep Blood Agar, and Brain Heart Infusion Agar under anaerobic conditions (90% hydrogen, 10% carbon dioxide) for 72 h. The researcher obtained pure colonies through subculturing before performing catalase and oxidase and Gram staining tests. The researchers used McFarland criteria to create bacterial suspensions in sterile saline before they inserted VITEK 2 cards (GP, GN, or ANC) for exact bacterial identification.

### Statistical analyses

A priori power analysis was performed using G*Power 3.1.9.7 software (Heinrich Heine University, Düsseldorf, Germany). A one-way ANOVA test was selected, with an alpha level of 0.05, power of 0.80, and an effect size of *f* = 0.471. The effect size was calculated based on a predicted mean difference of 0.5 units in periodontal indices and a standard deviation of 0.5, consistent with similar study designs reported in the literature. The analysis indicated that a minimum of 16 participants per group was required [26]. Therefore, the current sample size can be considered sufficient to detect clinically meaningful differences. Prior to inferential analysis, the Shapiro–Wilk test was used to assess normality, while homogeneity of variances was examined using Levene’s test. Since both assumptions were met (*p* > 0.05), parametric tests were deemed appropriate. The data were analyzed using SPSS software (IBM SPSS Statistics, Version 22) through one-way ANOVA and paired sample-T tests to evaluate within- and between-group differences. Statistically significant variances identified by one-way one-way analysis of variance (ANOVA) were further analyzed using Duncan’s multiple-range test. As the assumption of variance homogeneity was satisfied, Duncan’s multiple-range test was chosen to better detect intergroup differences. Although this test is statistically more liberal and may increase the risk of Type I error, it offers higher power in post-hoc comparisons. Future studies are encouraged to report effect sizes (e.g. eta-squared, Cohen’s d) and 95% confidence intervals in addition to *p*-values to enhance the interpretability and clinical relevance of the findings. In order to test intraobserver reliability, periodontal index measurements were repeated at two different time points in 15 individuals. No statistically significant differences were observed between the repeated measurements, and a high level of correlation was reported (*r* = 0.73–0.91). Additionally, Cohen’s kappa coefficients for ordinal-scale indices ranged from 0.70 to 0.79, indicating a high level of intraexaminer reliability. The results have been presented in charts, with significant changes indicated by different letter groupings.

## Results

Analysis of periodontal parameters as shown in Table 1 revealed that, at T1, the mean probing depth differed significantly among groups (*p* = 0.032), with higher values in the removable and fixed appliance groups compared with the control group, and a significant intragroup increase was observed only in the fixed appliance group (*p* = 0.003). Similarly, plaque index values showed a significant increase at T1 for both the removable (*p* = 0.019) and fixed appliance groups (*p* = 0.023). Regarding microbiological findings in Tables 2 and 3, a significant increase in yeast counts was detected in the removable appliance group (*p* = 0.008), whereas *Lactobacillus* counts significantly decreased in both the control (*p* = 0.005) and removable appliance groups (*p* = 0.004). In the fixed appliance group, *Lactococcus* levels increased significantly (*p* = 0.042). Additionally, mutans streptococci levels increased significantly in both the removable (*p* = 0.026) and fixed appliance groups (*p* = 0.037), while a significant decrease in total anaerobic microorganism counts was observed in the control group (*p* = 0.024).

**Table 1:** Probing depth, attachment level, plaque index, and bleeding on probing values were summarized by means across all time points among groups.

*Time*	Control group	Removable appliance	Fixed appliance	*F*	*P*
**Mean probing depth**					
**T0**	1.318 ± 0.867b	1.581 ± 0.813a	1.250 ± 0.456bB	5.662**	0.006
**T1**	1.338 ± 0.107b	1.700 ± 0.103a	1.538 ± 0.166abA	3.745*	0.032
***t***	−0.436	−1.623	−3.502*		
***p***	0.669	0.125	0.003		
**Mean attachment level**					
**T0**	1.644 ± 0.322	1.594 ± 0.558	1.475 ± 0.502	0.034	0.967
**T1**	1.563 ± 0.338	1.731 ± 0.553	1.581 ± 0.504	0.038	0.963
***t***	0.612	−0.702	−1.204		
***p***	0.549	0.493	0.247		
**Plaque index**					
**T0**	1.244 ± 0.140	1.538 ± 0.88B	1.300 ± 0.098B	1.972	0.152
**T1**	1.213 ± 0.165b	1.881 ± 0.148aA	1.700 ± 0.158aA	4.827*	0.013
***t***	0.628	−2.624*	−2.521*		
***p***	0.539	0.019	0.023		
**Percentage values of bleeding on probing**					
**T0**	21.438 ± 5.789	24.375 ± 3.760B	20.000 ± 2.604B	0.274	0.762
**T1**	19.288 ± 5.445	34.750 ± 5.460A	32.563 ± 4.650A	2.590	0.086
***t***	1.841	−2.592*	−2.855*		
***p***	0.086	0.020	0.012		

In each column (A, B ↓) and row (a, b, c →), different letters indicate statistically significant differences between groups and times at *p* < 0.05.

* *p* < 0.05.

** *p* < 0.001.

Values are presented as mean ± standard deviation.

**Table 2:** Comparison of microorganism counts (CFU-Log10) between different time points and appliance groups.

*Time*	Control group	Removable appliance	Fixed appliance	*F*	*p*
**Total aerobic mesophilic microorganism count CFU-(Log10)**					
**T0**	5.430 ± 0.162	5.547 ± 0.132	5.371 ± 0.137	0.383	0.685
**T1**	5.075 ± 0.187	5.290 ± 0.222	5.170 ± 0.156	0.324	0.727
***t***	1.771	1.177	0.921		
***p***	0.097	0.257	0.371		
**Total anaerobic microorganism count CFU-(Log10)**					
**T0**	5.226 ± 0.188A	4.575 ± 0.360	4.905 ± 0.366	1.07	0.353
**T1**	4.735 ± 0.135B	4.707 ± 0.213	4.561 ± 0.349	0.41	0.869
***t***	2.518*	−0.269	0.765		
***p***	0.024	0.792	0.456		
**Microaerophilic microorganism count CFU-(Log10)**					
**T0**	5.271 ± 0.176	5.601 ± 0.171	5.493 ± 0.087	1.25	0.296
**T1**	5.295 ± 0.138	5.394 ± 0.187	4.641 ± 0.474	1.80	0.176
***t***	−0.113	0.750	1.900		
***p***	0.911	0.465	0.077		

In each column, letters A and B (↓) indicate statistically significant differences between days at *p* < 0.05.

* : *p* < 0.05.

CFU: Colony-forming units.

Values are presented as mean ± standard deviation.

**Table 3:** Comparison of microorganism counts (CFU-Log10) for yeast and specific bacteria between different time points and appliance groups.

Time	Control group	Removable appliance	Fixed appliance	*F*	*p*
**Yeast count CFU-(Log10)**					
**T0**	0.669 ± 0.299	0.269 ± 0.184B	1.104 ± 0.332	2.24	0.118
**T1**	0.865 ± 0.237	1.069 ± 0.273A	1.747 ± 0.308	2.84	0.069
***t***	−0.453	−3.029*	−1.510		
***p***	0.657	0.008	0.152		
***Lactococcus* microorganism count CFU-(Log10)**					
**T0**	4.814 ± 0.258	4.735 ± 0.397	4.749 ± 0.210B	0.021	0.981
**T1**	4.778 ± 0.184	4.923 ± 0.250	5.338 ± 0.184A	1.942	0.155
***t***	0.095	−0.358	−2.222*		
***p***	0.926	0.725	0.042		
***Staphylococcus-Micrococcus* microorganism count CFU-(Log10)**					
**T0**	0.850 ± 0.216	0.333 ± 0.185	0.850 ± 0.227	2.032	0.143
**T1**	1.213 ± 0.207	0.861 ± 0.184	1.356 ± 0.252	1.390	0.259
***t***	−1.197	−1.792	−1.762		
***p***	0.250	0.093	0.098		
***Lactobacillus* count CFU-(Log10)**					
**T0**	5.381 ± 0.192A	5.666 ± 0.147A	5.117 ± 0.142	2.87	0.067
**T1**	4.465 ± 0.186B	4.401 ± 0.385B	4.838 ± 0.221	0.72	0.493
***t***	3.275*	3.379*	1.141		
***p***	0.005	0.004	0.272		
***Staphylococcus aureus* count CFU-(Log10)**					
**T0**	0.481 ± 0.224	0.829 ± 0.312	0.715 ± 0.225	0.474	0.625
**T1**	0.764 ± 0.215	0.375 ± 0.149	0.610 ± 0.245	0.896	0.415
***t***	−1.238	1.131	0.480		
***p***	0.235	0.276	0.638		
**Mutans streptococci count CFU-(Log10)**					
**T0**	4.873 ± 0.186b	5.718 ± 0.136aA	4.799 ± 0.137bB	4.553*	0.016
**T1**	4.950 ± 0.203	5.133 ± 0.247B	5.453 ± 0.166A	1.494	0.235
***t***	−0.403	2.479*	−2.289*		
***p***	0.693	0.026	0.037		

In each column (A, B ↓) and row (a, b, c →), different letters indicate statistically significant differences between groups and times at *p* < 0.05.

* : *p* < 0.05.

CFU: Colony-forming units.

Values are presented as mean ± standard deviation.

**Figure 3:**
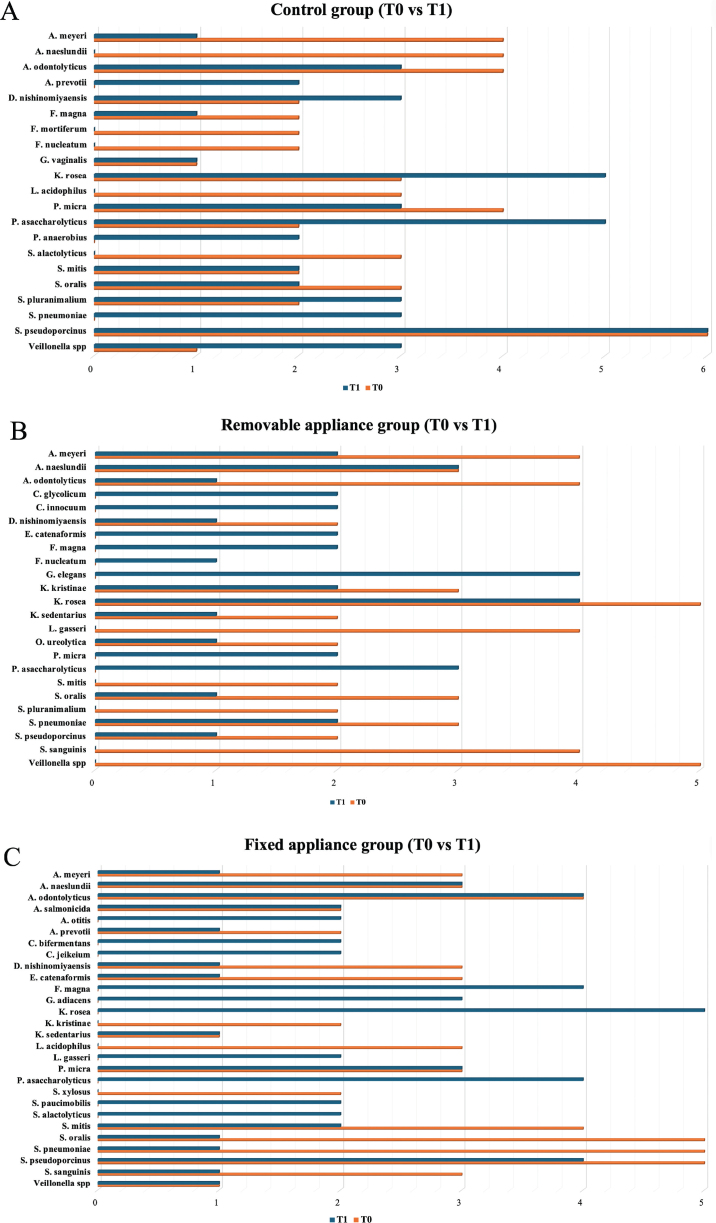
Microorganism changes between groups at T0 and T1; control group (A), removable appliance group (B), and fixed appliance group (C) (microorganisms detected only once across T0 and T1 were omitted from the figure.)

Ninety-six samples were collected in this study, and 352 VITEK 2 cards were used for analysis. However, 10 cards were excluded from the evaluation due to low identification accuracy or insufficient sample volume. In the control group, 21 microorganisms were identified using 54 VITEK 2 cards during the T0 period, while 26 were identified using 57 VITEK 2 cards during the T1 period. In the removable appliance group, 18 microorganisms were identified using 55 VITEK 2 cards during the T0 period, and 34 microorganisms were identified using the same number of cards during the T1 period. In the fixed appliance group, 21 microorganisms were identified using 57 VITEK 2 cards during the T0 period, and 34 microorganisms were identified using 64 VITEK 2 cards during the T1 period. The increase in microbial diversity within the orthodontic treatment groups is detailed in Figure 3.

## Discussion

Orthodontic patients need both clinical evaluations and microbiological tests to determine their periodontal health because this data enable doctors to develop suitable treatment plans and assess treatment outcomes. The two orthodontic treatment approaches of fixed and removable appliances operate as separate systems that produce distinct effects on dental health.

In our study, the absence of mobility and furcation findings was associated with the contraindication of orthodontic treatment in individuals with active periodontal problems. The immobility observed during the T1 period was attributed to applying light forces, the absence of primary occlusal contacts, and the health of periodontal tissues. Data from longitudinal clinical studies [27, 28] suggest that orthodontic treatment with fixed appliances has a negligible effect on periodontal attachment levels. In our study, the mean probing depth in the fixed appliance group was statistically significant at the 6th month post-treatment. These findings are consistent with those of several other researchers. Our findings indicate a nonsignificant gain in attachment levels in the removable and fixed treatment groups at the 6th month of treatment. While our study aligns with findings reported in previous studies [29, 30]. This discrepancy stems from the age-based analysis conducted in those studies.

Our study observed a statistically significant increase in plaque index at the T1 time point compared to the T0 time point in the orthodontic treatment groups. These findings are consistent with changes in plaque index reported in previous studies [31–33]. Additionally, a significant increase in bleeding on probing percentage was detected in both treatment groups from T0 to T1. These results emphasize the need for improved oral hygiene practices and align with findings from other studies [34, 35].

Upon evaluation of the yeast-related parameters, our study demonstrated an increase in yeast counts within the orthodontic treatment groups. Studies on the effect of orthodontic treatments on Candida colonization [34, 36] have reported results consistent with our findings.

*Lactococcus* is a bacterium commonly found in the oral flora and associated with the fermentation of dairy products. A literature review revealed no studies reporting the potential effects of orthodontic treatments on *Lactococcus* bacterial species. This study observed an increase in *Lactococcus* microorganisms from T0 to T1 in orthodontic treatment groups. The observed increase in this microorganism during the T1 period in orthodontic treatment groups could be attributed to changes in the patient’s dietary habits.

Research studies [37, 38] have investigated *Lactobacillus* species that demonstrate strong cariogenic properties during orthodontic treatment of patients. The research demonstrated that all three groups showed a major decrease in their *Lactobacillus* microorganism populations. The combination of improved oral hygiene and higher fluoride toothpaste usage and dietary changes led to decreased microorganism numbers.

In the literature [39], *S. mutans* is described as a bacterium with cariogenic effects, and for this reason, a study [40] has been conducted regarding changes in *S. mutans* levels in patients undergoing fixed orthodontic treatment. According to the results of our study, a statistically significant increase (*p* < 0.05) in the number of *S. mutans* was observed in the fixed orthodontic treatment group. This finding is consistent with the literature [41, 42]. On the other hand, in the group using removable appliances, a decrease in microorganism count was detected at T1. This reduction could be attributed to the fact that individuals can remove their removable appliances during eating and drinking, allowing for more regular oral hygiene.

Although no statistically significant periodontal changes were observed within the control group across the follow-up period, a noteworthy reduction was detected in both *Lactobacillus* counts and total anaerobic microorganism levels. The decrease in microbial numbers could result from typical changes in personal oral bacterial populations or participants enhancing their oral care practices because of research participation (Hawthorne effect) or unmonitored changes in their diet and daily routines.

Orthodontic appliances can modify the oral microbiota by increasing plaque accumulation, potentially facilitating the growth of pathogenic species. However, not all microorganisms that increase during treatment are harmful. Therefore, identifying specific bacterial subgroups is essential to assess periodontal risk, as no single species has been definitively linked to active periodontal disease, though some serve as risk indicators. In our study, similar microorganisms were identified among the groups during the T0 period, with common species including *A. meyeri, A. naeslundii, A. odontolyticus, D. nishinomiyaensis, K. rosea, L. acidophilus, P. micra, S. mitis, S. oralis, S. pseudoporcinus, S. sanguinis,* and *Veillonella spp.*

The microorganisms with high pathogenic potential identified during the T1 period include A. otitis, C. butyricum, C. clostridioforme, C. glycolicum, C. histolyticum, E. faecalis, E. rhusiopathiae, F. nucleatum, G. elegans, S. aureus, P. micra, P. anaerobius, P. oralis, and S. pneumoniae. The presence of A. meyeri, A. naeslundii, and A. odontolyticus species in samples indicates their role as primary pathogens that cause infections in different parts of the body [43]. The detection of these bacteria could indicate that oral aphthous ulcers existed at the time of sample collection.

The anaerobic microorganism *Lactobacillus acidophilus* is most commonly found in humans, particularly in the gastrointestinal tract and oral cavity [44]. Its frequent detection in this study is likely attributed to the oral cavity being its natural habitat.

*Parvimonas micra* is an anaerobic microorganism frequently isolated from dental plaque in patients with chronic periodontitis [45]. It was commonly detected in samples collected during the T0 and T1 periods. As one of the bacteria in biofilm formation within the oral cavity, *P. micra* benefits from the biofilm structure, facilitating its persistence in periodontal pockets.

*S. mitis, S. oralis, S. pseudoporcinus*, and *S. sanguinis* species were isolated during the T0 and T1 periods. These streptococcal species are known as primary commensals of the oral cavity. They exhibit antagonistic effects against pathogens such as *S. mutans* and *P. gingivalis*, thereby contributing positively to the oral microbiota [46].

*F. magna* is an anaerobic coccus with a high pathogenic potential [47] and is part of the normal human mucocutaneous flora and has been isolated in deep organ abscesses, obstetric and gynecological sepsis, oral infections, and sinusitis [48]. In our study, this microorganism was frequently detected during the T1 period, which may indicate the presence of an oral infection at that time.

During the T1 period, the isolation of pathogenic microorganisms suggests possible ongoing infections such as otitis, sinusitis, or tonsillitis. Although these species are not primary pathogens, the anaerobic environment during infection may promote their growth. Prior study [49] has shown that fixed orthodontic appliances can alter supragingival and salivary microflora, favoring facultative aero-anaerobic species. In our study, a microbial shift from aerobic to anaerobic species was observed from T0 to T1, potentially due to dietary or oral hygiene changes that lower pH and promote anaerobic proliferation by creating protected ecological niches.

When the periodontal and microbiological findings are interpreted together, a clearer pattern emerges regarding the biological impact of orthodontic appliances. The increase in plaque index, probing depth, and bleeding on probing in both treatment groups coincided with a shift in the microbial profile, characterized by reduced aerobic and microaerophilic counts and a relative predominance of anaerobic species. In the fixed appliance group, the rise in *S. mutans* levels is consistent with the greater plaque accumulation and bleeding scores, supporting its role as a cariogenic and plaque-associated pathogen under conditions of increased biofilm retention. By contrast, in the removable appliance group, the decrease in *S. mutans* and *Lactobacillus* counts, together with less pronounced periodontal changes, may reflect the advantage of being able to remove the appliance during oral hygiene procedures. The concurrent increase in *Lactococcus* and *Staphylococcus–Micrococcus* species likely represents ecological adaptation to new retention surfaces rather than overt disease, but these shifts may still signal a higher susceptibility to periodontal imbalance in patients with inadequate plaque control.

This study possesses several methodological strengths that enhance the reliability, novelty, and interpretability of its findings. The inclusion of a nontreatment control group allowed for valid temporal comparisons and helped differentiate appliance-related alterations from natural microbial or periodontal fluctuations. Furthermore, the species-level microbial identification achieved through the VITEK 2-automated system represents a novel methodological contribution to orthodontic research, providing higher diagnostic precision than conventional culture-based approaches. The use of standardized periodontal indices, two-time-point sampling, and consistent sampling procedures further contributed to internal validity. Moreover, evaluating both fixed and removable orthodontic appliances within the same research framework allowed for direct comparison of their biological effects, thereby offering a broader clinical perspective.

This study should be interpreted considering several limitations. First, the follow-up period was limited to 6 months, which restricts the ability to determine long-term periodontal and microbial alterations; therefore, future studies incorporating post-treatment and retention-phase assessments are warranted. Second, the use of conventional culture-based microbiological techniques may have limited detection to cultivable organisms only, potentially overlooking uncultivable or low-abundance species. Additionally, variations in participants’ oral hygiene practices and dietary habits were not controlled during the study period, which may have influenced microbial composition. As all periodontal measurements were performed by a single examiner, inter-examiner reliability could not be assessed, and this has been acknowledged as an additional limitation. Third, the use of a single diagnostic platform (VITEK 2) without molecular confirmation may restrict the depth of microbial characterization. Future studies should therefore consider larger sample sizes, multicenter designs, molecular-based microbial analysis, and extended follow-up durations.

Future studies should include longer follow-up periods extending into the post-treatment and retention phases to clarify long-term microbial and periodontal outcomes. Additionally, molecular-based identification techniques such as 16S rRNA sequencing, qPCR, or metagenomic profiling should be employed to detect noncultivable and low-abundance microorganisms with higher precision. Standardization of oral hygiene routines and diet-control protocols, ideally through patient diaries or digital tracking, would minimize behavioral confounders. Finally, multicenter trials with larger and more diverse patient populations are needed to enhance the external validity of the findings and facilitate subgroup analyses based on age, appliance type, and oral hygiene level. Beyond research implications, clinical practice should also be considered. From a clinical perspective, periodic periodontal co-management through scheduled referrals to periodontologists may optimize patient motivation and compliance, facilitate timely detection of early periodontal alterations, and ultimately support the maintenance of periodontal health and long-term treatment stability, particularly throughout the retention period.

The research findings support the partial acceptance of the study hypothesis because fixed appliances produced worse results in particular periodontal and microbial parameters, but other variables showed no significant differences.

## Conclusions

The research findings showed that orthodontic appliances with fixed and removable designs create measurable changes in periodontal health and microbial populations. The research results emerged from the established study parameters, which produced the following findings:

- Plaque index, probing depth, and bleeding on probing values increased at 6 months in both treatment groups, indicating heightened periodontal susceptibility during active appliance use.
- Aerobic mesophilic and microaerophilic microorganism counts decreased, while anaerobic counts demonstrated appliance-dependent directional variation, suggesting an ecological shift toward reduced oxygen-tolerant species.
- The microbial community showed different responses to appliances through *S. mutans* population growth in fixed appliance users and decline in removable appliance users.
- The yeast population increased while *Lactobacillus* and *S. aureus* numbers decreased in all treatment groups, which showed unpredictable effects on caries-causing microorganisms and opportunistic pathogens.
- The microbial community contained higher numbers of *Lactococcus* and *Staphylococcus–Micrococcus* species because of surface alterations caused by appliances and dietary components.
- The pathogenic potential of isolated species from both appliance types proved equal, which shows microbial changes stem from ecological shifts instead of disease progression.

## Supplementary material

The data that support the funding of this study are available from the corresponding author upon reasonable request.
