# Corrigendum: Microbial and periodontal shifts induced by orthodontic appliances: a comparative clinical study

**DOI:** 10.2340/aos.v85.47168

**Published:** 2026-11-05

The corresponding author of the article: Atasever İşler AA, Erdem A. *Microbial and periodontal shifts induced by orthodontic appliances: a comparative clinical study,* requested a corrigendum to add two pieces of information that were unintentionally omitted from the published article:

## Original text

### Acknowledgments

The authors declare that this study was financially supported by Atatürk University Scientific Research Projects (TDK2022/10130). We sincerely thank the Department of Food Hygiene and Technology at Atatürk University Faculty of Veterinary Medicine for granting us access to their microbiology laboratory. We would like to express our sincere gratitude to our project coordinator, Prof. Dr. Nihat Kılıç, for his valuable contributions and support throughout the course of this project.

## New text

### Acknowledgments

This article is based on the doctoral thesis of Aybüke Asena Atasever İşler, entitled ‘The Effect of Orthodontic Treatment Applications on Oral Flora and Evaluation of Individuals’ Knowledge and Attitudes About Oral Hygiene’, conducted under the supervision of Prof. Dr. Abdulvahit Erdem (Atatürk University, Institute of Health Sciences, Erzurum, Türkiye, 2023; Thesis No: 857113).

The authors declare that this study was financially supported by Atatürk University Scientific Research Projects (TDK2022/10130). We sincerely thank the Department of Food Hygiene and Technology at Atatürk University Faculty of Veterinary Medicine for granting us access to their microbiology laboratory. We would like to express our sincere gratitude to our project coordinator, Prof. Dr. Nihat Kılıç, for his valuable contributions and support throughout the course of this project.

## Original text (in the Discussion section)

The microorganisms with high pathogenic potential identified during the T1 period include *A. otitis, C. butyricum, C. clostridioforme, C. glycolicum, C. histolyticum, E. faecalis, E. rhusiopathiae, F. nucleatum, G. elegans, S. aureus, P. micra, P. anaerobius, P. oralis*, and *S. pneumoniae*. The presence of *A. meyeri, A. naeslundii,* and *A. odontolyticus* species in samples indicates their role as primary pathogens that cause infections in different parts of the body [43]. The detection of these bacteria could indicate that oral aphthous ulcers existed at the time of sample collection.

## New text

The microorganisms with high pathogenic potential identified during the T1 period include *A. otitis, C. butyricum, C. clostridioforme, C. glycolicum, C. histolyticum, E. faecalis, E. rhusiopathiae, F. nucleatum, G. elegans, S. aureus, P. micra, P. anaerobius, P. oralis*, and *S. pneumoniae*. The presence of *A. meyeri, A. naeslundii,* and *A. odontolyticus* species in samples indicates their role as primary pathogens that cause infections in different parts of the body [43]. The Actinomyces species in this cohort were reported in [50]. The detection of these bacteria could indicate that oral aphthous ulcers existed at the time of sample collection.
